# Determination of CO_2_ Solubility in Water by NIR Spectroscopy under Different Temperature and Pressure Conditions

**DOI:** 10.1021/acsomega.5c07060

**Published:** 2025-11-28

**Authors:** Lorena Armando da Silveira, Lorena Mariah Oliveira Lima, Thiago Rodrigues da Cunha, Fabiane Santos Serpa, Ranyere Lucena Souza, Rosane Alves Fontes, Giselle Maria Lopes Leite da Silva, Mônica Teixeira da Silva, Jussara de Mello Silva, Tiago Cavalcante Freitas, Luiz Alexandre Sacorague, Claudio Dariva, Marcos Lúcio Corazza, Elton Franceschi

**Affiliations:** † Center for Studies on Colloidal Systems, Institute of Technology and Research, 67896 Tiradentes University, Av. Murilo Dantas, 300, Aracaju, SE 49032-490, Brazil; ‡ Chemical Engineering Department, 28122 Federal University of Parana, Rua Coronel Francisco Heráclito dos Santos, 210, Curitiba, PR 81531-990, Brazil; § Research Center Leopoldo Américo Miguez de Mello, 125096 Petróleo Brasileiro SA, Av. Horácio Macedo, 950, Ilha do Fundão, Rio de Janeiro, RJ 20031-912, Brazil

## Abstract

Monitoring the solubility of carbon dioxide (CO_2_) in water under high-pressure and -temperature conditions is critical for industrial processes. Although the solubility behavior of CO_2_ is well-established in the literature, most conventional methods rely on offline, invasive, or indirect measurements. In this study, a methodology was developed to monitor CO_2_ solubility in water using near-infrared (NIR) spectroscopy due to its sensitivity to molecular interactions and ability to detect structural changes in the aqueous matrix in real time. The experimental system consisted of an apparatus similar to the static synthetic method coupled with an NIR spectrophotometer, enabling noninvasive spectral data acquisition during the dissolution of CO_2_ in ultrapure water under varying temperature (40 to 60 °C) and pressure (0.15 to 2.35 MPa) conditions. Spectral data were preprocessed and correlated with CO_2_ solubility using partial least-squares (PLS) regression. The PLS model was calibrated using solubility values obtained from a thermodynamic model based on the Peng–Robinson equation of state (PR-EoS) and the NRTL activity coefficient model, which was parametrized using experimental data from the literature. The developed model showed high predictive performance (*R* ^2^ > 0.99) with an average external prediction error of 9.91%. In comparison, the thermodynamic model yielded a mean absolute deviation of 0.073% in pressure predictions. The results demonstrate that NIR spectroscopy is sensitive to spectral variations associated with molecular interactions between CO_2_ and water, enabling the construction of robust predictive models. This integrated approach provides a reliable and real-time method for indirectly estimating CO_2_ solubility from spectral data. It thus represents an alternative tool for industrial and environmental monitoring of dissolved gases.

## Graphical abstract

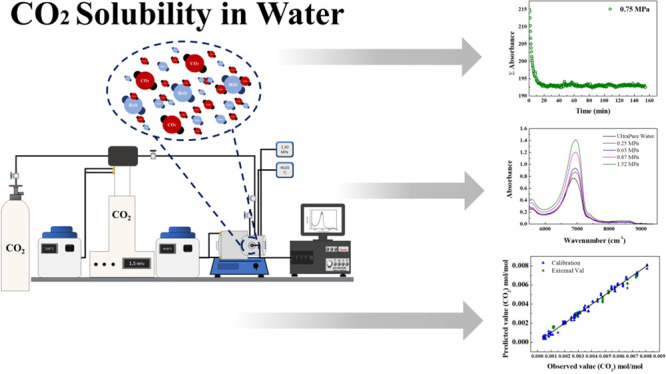

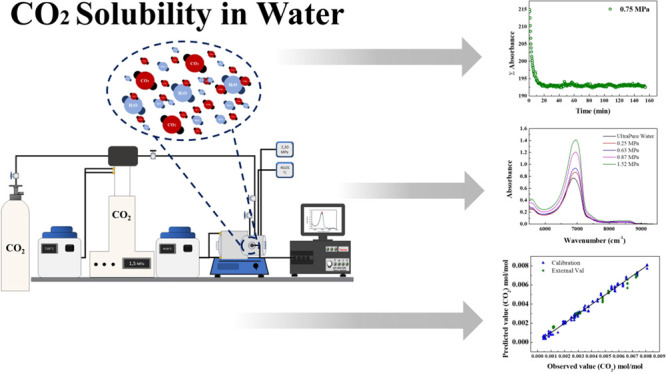

## 1 Introduction

The solubility of gases in liquids has been extensively investigated over the past decades due to its broad relevance in industrial and environmental applications, such as enhanced oil recovery (EOR), gas absorption, and the mitigation of phenomena like scaling and corrosion.^1–3^ Among the systems of greatest practical interest is the dissolution of CO_2_ in water, which plays a key role in processes involving gas–liquid mass transfer.^1,2,4–12^ It is well-known that the solubility of CO_2_ in water increases with increasing pressure and decreases with increasing temperature, and its behavior has been extensively characterized under equilibrium conditions.

The methods traditionally employed to determine CO_2_ solubility include the synthetic method, the volumetric method, and offline analytical techniques such as chromatography and titration. Although widely used, these approaches present limitations, such as their invasive nature, the requirement of sample withdrawal, low temporal resolution, and restrictions under conditions of low solubility. Fourier-transform infrared (FTIR) spectroscopy, in turn, enables the direct detection of CO_2_ via its asymmetric stretching band at approximately 2340 cm^–1^ and bending mode at 660 cm^–1^. However, its application in pressurized aqueous systems is severely constrained by the strong absorption of water in the mid-infrared (MIR), which saturates the signal and necessitates extremely short optical path cells, making in situ measurements difficult.^13,14^

In this context, near-infrared (NIR) spectroscopy emerges as an alternative tool for noninvasive and real-time monitoring of gas–liquid systems.^3,15,16^ Although NIR spectroscopy provides an indirect measurement since the spectral changes arise from perturbations in water’s O–H vibrational modes rather than from direct CO_2_ absorption it offers decisive advantages.^17^ By operating in a region dominated by weaker overtones and combination bands, NIR allows the use of probes with longer optical paths (1–20 mm) and enables continuous and noninvasive monitoring under pressurized conditions. These spectral changes, associated with rearrangements in the hydrogen-bond network of water induced by dissolved CO_2_, can be reliably correlated with the degree of CO_2_ solubility, thereby overcoming the limitations of FTIR for aqueous pressurized systems and offering clear advantages for industrial applications.

Unlike traditional methods, NIR spectroscopy enables continuous monitoring of the system without the need for sample collection or manipulation, making it especially suitable under extreme pressure and temperature conditions.^3^ Although previous studies have explored the application of NIR spectroscopy in assessing the solubility of water in CO_2_-rich systems, few have directly employed NIR to evaluate the solubility of CO_2_ in water, particularly under high-pressure and high-temperature conditions. Previous works, such as those by Muncan et al.,^15^ Jackson et al.,^16^ and Torres et al.,^3^ primarily focused on the spectral characterization of water under supercritical CO_2_ conditions, without directly addressing the spectroscopic monitoring of gas dissolution into the aqueous phase.

Therefore, this study aimed to investigate the applicability of NIR spectroscopy as a tool to monitor CO_2_ solubility in water under varying temperature and pressure conditions. By exploring the spectral behavior of the aqueous matrix during the CO_2_ dissolution process, this work seeks to contribute to the development of continuous monitoring methods with potential applications in the oil and gas industry, carbon capture technologies, and environmental process control.

## 2 Methodology

### 2.1 Materials

All experimental measurements were carried out using ultrapure, double-distilled and deionized, water (resistivity of 18.2 MΩ·cm) and carbon dioxide (CO_2_) with a purity of 99.9%, supplied by White Martins S.A.

### 2.2 Experimental System and Procedure

Figure 1 illustrates the experimental apparatus used to obtain CO_2_ solubility measurements in water, conducted under varying temperature (40 to 60 °C) and pressure (0.15 to 2.35 MPa) conditions using NIR spectroscopy. Solubility data were obtained using a method similar to the static synthetic phase equilibrium method. The system comprises a CO_2_ cylinder [1], connected to a ball valve [2] that regulates the gas flow into a syringe pump [3] (260D, TELEDYNE (ISCO)), coupled with a cooling bath [4] to maintain the thermal stability of the gas during transfer. After transferring the gas to the pump, the cylinder is disconnected from the system. CO_2_ is transferred from the pump through a ball valve [5], which allows the fluid to inlet to the feed line, and a needle valve [6] enables precise flow control of CO_2_ to the equilibrium cell.

**Figure 1:**
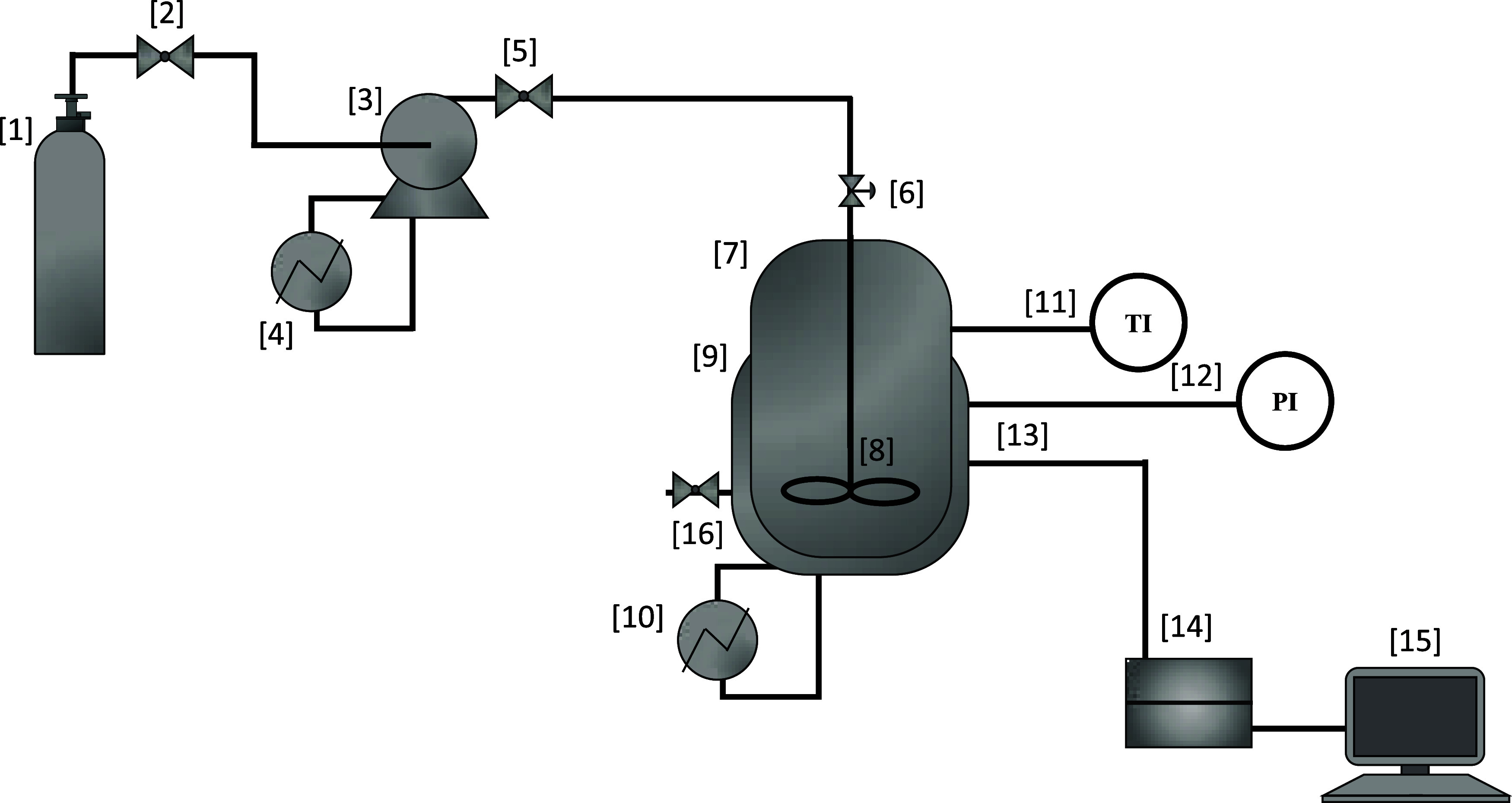
Bench-top unit for CO_2_ solubility measurements in water. [1] Carbon dioxide cylinder, [2] ball valve, [3] syringe pump, [4] thermostatic bath, [5] ball valve, [6] needle valve, [7] equilibrium cell, [8] magnetic stirring plate, [9] aluminum heating/cooling block, [10] thermostatic bath, [11] temperature indicator, [12] pressure transducer, [13] NIR probe, [14] spectrophotometer, [15] computer, and [16] ball valve.

The equilibrium cell [7], made of stainless steel, includes a continuous stirring system composed of a magnetic stirrer plate and a magnetic bar [8] placed inside the cell. Temperature control is achieved using a jacketed aluminum block [9] through which thermal fluid from a thermostatic bath [10] (TECNAL, TE-184) circulates. Pressure and temperature inside the cell were continuously monitored using a pressure transducer [12] (YOKOGAWA, EJA530E) and a type J thermocouple [11] (SALCAS), respectively, both connected to digital process indicators (NOVUS, N1500). A NIR probe [13] was inserted into the cell and connected to a spectrophotometer [14] (Thermo Fisher Scientific, Antaris MX), equipped with a tungsten-halogen lamp, ensuring continuous emission across the near-infrared region, which transmitted spectral data to a computer [15]. The probe geometry was of parallel fixed path type, allowing internal reflection with controlled alignment to minimize optical losses and to maintain reproducibility of measurements under pressurized liquid conditions. All tubing and connections were made of 1/8″ external diameter of stainless steel, ensuring chemical compatibility and mechanical strength. A secondary ball valve [16] was installed downstream to allow for rapid depressurization, serving as a safety mechanism for operational discharge.

Ultrapure water was degassed using an ultrasonic bath until visible gas bubbles were completely removed, and 30 mL were added to the equilibrium cell. While maintaining the system at a constant temperature, CO_2_ was carefully injected through the feed valve [6] until reaching the first target pressure to be monitored. At this point, the acquisition of NIR absorption spectra was initiated to monitor the liquid–vapor phase equilibrium. To a new pressure measurement, more CO_2_ was injected into the equilibrium cell. The Figure 2 provide a detailed arrangement illustration of the NIR probe [13], light source, aperture, and sapphire window geometry [17], used only to visualization of the system, complementing the overall experimental setup depicted in Figure 1. This schematic highlights the optical path and key structural elements affecting the precision of spectral acquisition.

**Figure 2:**
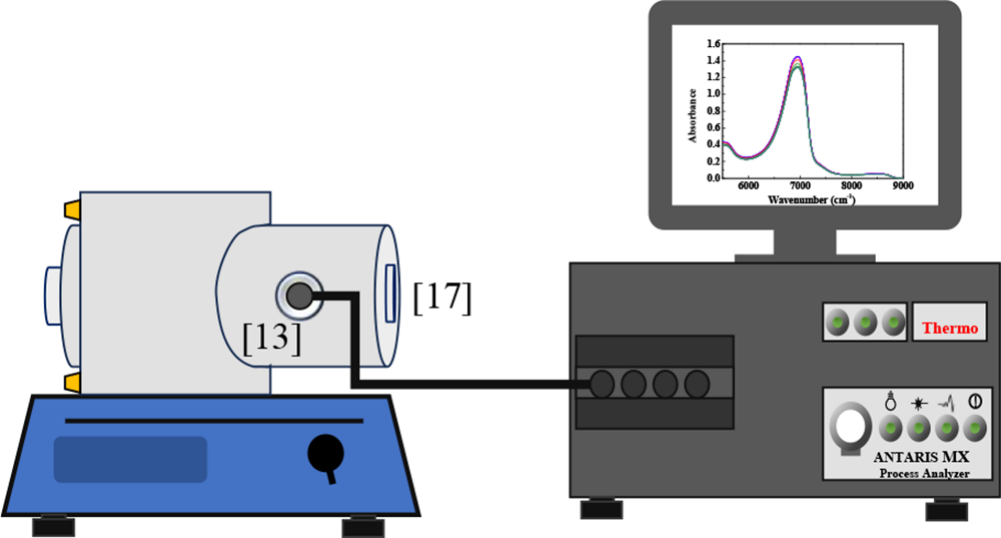
Schematic representation of the NIR optical system coupled to the balance cell. The diagram shows the NIR probe [13] and the sapphire window used to seal the cell [17].

### 2.3 Acquisition and Processing of NIR Spectra

Spectral measurements were performed using the spectrophotometer coupled with a 1 mm transflectance probe (650 series, Precision Sensing Devices, PSD Inc.), operating in the range of 5500 to 10,000 cm^–1^ with a resolution of 8 cm^–1^. Each spectrum consisted of 32 scans controlled by the RESULT 3 software. Spectra were continuously acquired during the dissolution of CO_2_, with data collected every 19 s until the vapor–liquid equilibrium was reached. The system was considered to have reached equilibrium when the pressure and the absorbance sum, in the 5901.128 to 7999.28 cm^–1^ range, remained constant for at least 1 h. This range includes bands sensitive to CO_2_ in water interactions. A total of 16,095 spectra were acquired, of which 3030 corresponded to equilibrium conditions (used for model calibration) and 809 were used for external validation.

### 2.4 Calibration of the Monitoring Model

To correlate the NIR spectra with CO_2_ solubility, a multivariate calibration model was developed using partial least-squares (PLS) regression. The purpose of using this model was to extract relevant latent variables from the high-dimensional spectral data that best explained the variance in CO_2_ concentration, even in the presence of collinearity. This approach allows for the construction of a predictive model capable of estimating dissolved CO_2_ directly from spectral features.

In this study, the term calibration refers specifically to the subset of spectra employed to train the PLS model and to define the mathematical relationship between spectral variables (*x*) and dissolved CO_2_ concentration (*y*). The validation set, in contrast, consisted of independent spectra used exclusively to assess the predictive accuracy and generalization capability of the model. Two data sets were defined: one for calibration (60–80% of spectra) and another for external validation (20–40%), both randomly partitioned to preserve statistical representativeness.^3^

The absorbance was considered not as a single integrated value, but as a spectral vector within the range 5500–9500 cm^–1^, which encompasses the first overtone and combination bands of water O–H vibrations. This spectral region is particularly sensitive to structural perturbations in the hydrogen-bond network of water induced by dissolved CO_2_. Prior to modeling, spectral preprocessing included vector normalization, applied to correct baseline fluctuations, and Savitzky-Golay smoothing (15-point window, second-order polynomial), applied to attenuate high-frequency noise and enhance signal quality.^17^

The reference values of dissolved CO_2_ concentration at the equilibrium were not obtained directly from the spectra but from a thermodynamic γ-ϕ model, parametrized with experimental literature data. This model provided equilibrium CO_2_ mole fractions as a function of the experimental temperature and pressure conditions. These values served as response variables (*y*) in the PLS regression, while the preprocessed spectra were used as predictor variables (*x*). The PLS model thus established a correlation between the NIR spectral variations in the 5500–9500 cm^–1^ region and the thermodynamically calculated CO_2_ solubility.^3,17^

The model was developed in Python language, and various segmentations and parametrizations were tested to assess sensitivity and robustness. Model performance was evaluated using the following statistical metrics: RMSE (root-mean-square error), *R* ^2^ (coefficient of determination), and Er (%) (mean relative error), according to the eqs 1–3.

$$\mathrm{RMSE}=\sqrt{\frac{\sum_{j=1}^{n}(y_{j},\mathrm{NIR}-y_{j},\exp{)}^{2}}{n}}$$ 1

$$R^{2}=\frac{\sum_{j=1}^{n}(y_{j},\mathrm{NIR}-\overset{-}{y},\exp{)}^{2}}{\sum_{j=1}^{n}(y_{j},\exp-\overset{-}{y},\exp{)}^{2}}$$ 2

$$\mathrm{Er}(\%)=\left[\frac{\sum_{j=1}^{n}\left(\frac{\left|y_{j},\mathrm{NIR}-y_{j},\exp\right|}{y_{j},\exp}\right)}{n}\right]\times 100$$ 3

Where *n* is the number of samples evaluated, *y*_*j*_, NIR represents the predicted values from the model, *y*_*j*_, EXP are the experimentally obtained values, and $\overset{-}{y},\exp$ is the mean of the experimental values. The optimal number of latent components was selected based on the root-mean-square error of cross-validation, supported by graphical inspection of predicted vs observed values to avoid underfitting or overfitting.

### 2.5 Thermodynamic Modeling of CO_2_ Solubility in Water

The solubility of CO_2_ in water was estimated, combining the Peng–Robinson equation of state (PR-EoS)^18^ for the vapor phase with the Non-Random Two Liquid (NRTL) activity coefficient model for the liquid phase.^3,19^

Vapor–liquid equilibrium modeling was conducted based on the isofugacity criterion, assuming low CO_2_ solubility. For this, Henry’s Law was applied (eq 4).

$$y_{i}{\hat{\varphi }}_{i}^{v}P=x_{i}H_{i}$$ 4

Where *x*_*i*_ and *y*_*i*_ are the mole fractions of component *i* in the vapor and liquid phases, φ̂_*i*_^*v*^ is the fugacity coefficient in the vapor phase, *P* is the system pressure, and *H*_*i*_ is Henry’s constant.

The vapor phase was described by the PR-EoS, with parameters obtained from the critical properties of the substances and van der Waals mixing rules for binary interactions (*k*_*ij*_ and *l*_*ij*_).^20^

For the liquid phase, the NRTL model was adopted for the calculation of activity coefficients and excess Gibbs energy (eqs 5 and 6).

$$\frac{{\underset{\_}{G}}^{\mathrm{ex}}}{\mathrm{RT}}=x_{i}x_{j}\left[\frac{\tau_{ji}{\underset{\_}{G}}_{ji}}{x_{i}+x_{j}G_{\mathrm{ji}}}+\frac{\tau_{ji}{\underset{\_}{G}}_{ij}}{x_{j}+x_{i}G_{ij}}\right]$$ 5

$$\ln\;\gamma_{i}=x_{j}^{2}\left[\tau_{ji}{\left(\frac{{\underset{\_}{G}}_{ji}}{x_{i}+x_{j}G_{ji}}\right)}^{2}+\frac{\tau_{ij}{\underset{\_}{G}}_{ij}}{(x_{j}+x_{i}{\underset{\_}{G}}_{ij}{)}^{2}}\right]$$ 6

The binary interaction parameters (τ_ij_, *G*_ij_) were fitted by minimizing the objective function (OF) based on the least-squares method, represented by eq 7:

$$\mathrm{OF}=\sum_{i=1}^{n_{\mathrm{obs}}}(P_{i}^{\exp}-P_{i}^{\mathrm{cal}}{)}^{2}$$ 7

Thus, the solubility of CO_2_ in the aqueous phase was calculated from the γ-ϕ approach, combining the PR-EoS for the vapor phase and the NRTL model for the liquid phase. The equilibrium condition was described by the equality of fugacity (eq 4). From this relation, the solubility values mole fraction of dissolved CO_2_ were obtained as a function of pressure and temperature. The calculated data were used as reference values to calibrate the chemometric model PLS, in which the calibration curve was constructed by directly relating the preprocessed NIR spectra with the CO_2_ concentrations predicted by the thermodynamic model.

For model parametrization, a database was compiled containing experimental results from 22 literature studies,^1,4–6,9–12,20–33^ covering temperatures from 1 to 175 °C and pressures up to 52.4 MPa (Supplementary Data). Based on the γ-ϕ approach, the model was fitted to the compiled data set by integrating solubility information with spectroscopic data. In this way, the solubility points determined in this study were correlated with the NIR spectra obtained under equilibrium conditions, reinforcing the analysis and validation of the model.

This modeling approach enabled the accurate estimation of CO_2_ solubility in the liquid phase under varying conditions. The solubility values obtained from the thermodynamic model were considered as reference experimental data and were subsequently used to feed the NIR-based chemometric model, allowing a quantitative correlation between the thermodynamic predictions and the spectral data. The critical properties and interaction parameters used for water and carbon dioxide in the thermodynamic model are presented in Table 1.

**Table 1:** Critical Properties and Interaction Parameters Used in the Thermodynamic Model

compound	*T*_c_ (°C)	*P*_c_ (MPa)	ω	sources
CO_2_ (1)	31.06	7.377	0.224	Hou et al.^1^
H_2_O (2)	374.15	22.120	0.344	Atkins et al.^2^

interaction parameters					
Peng–Robinson parameters		NRTL parameters			
*k*_ij_	*l_ij_*	*A*_12_	*A*_21_	*B*_12_	*B*_21_
0.0	0.0	–1.8984	6.13032	73.50022	–812.04808

## 3 Results and Discussion

Experimental measurements of CO_2_ solubility in water were conducted using an approach similar to the static synthetic method and monitored via NIR spectroscopy at temperatures of 40 to 60 °C and pressures ranging from 0.15 to 2.35 MPa. The upper limit of 2.35 MPa was defined by the safety specifications of the equilibrium cell and by the operational range of the NIR probe (up to 3.5 MPa). Although this pressure is below industrially relevant conditions greater than 7 MPa, near the supercritical state, the studied range was sufficient to validate the methodology and demonstrate its applicability. Future work may extend the methodology to higher pressures through the use of reinforced cells and suitable probes, thereby increasing its relevance to industrial contexts.

Figure 3 shows the variation in absorbance as a function of time during the dissolution process of CO_2_ in water at 40 °C. A progressive decrease in absorbance was observed throughout the dissolution process, indicating the incorporation of CO_2_ into the aqueous phase and the progression of the dissolution. The initial injection of CO_2_ at a pressure of 1.01 MPa resulted in a gradual pressure drop until stabilization at 0.75 MPa. This approach was used in all solubility points measured.

**Figure 3:**
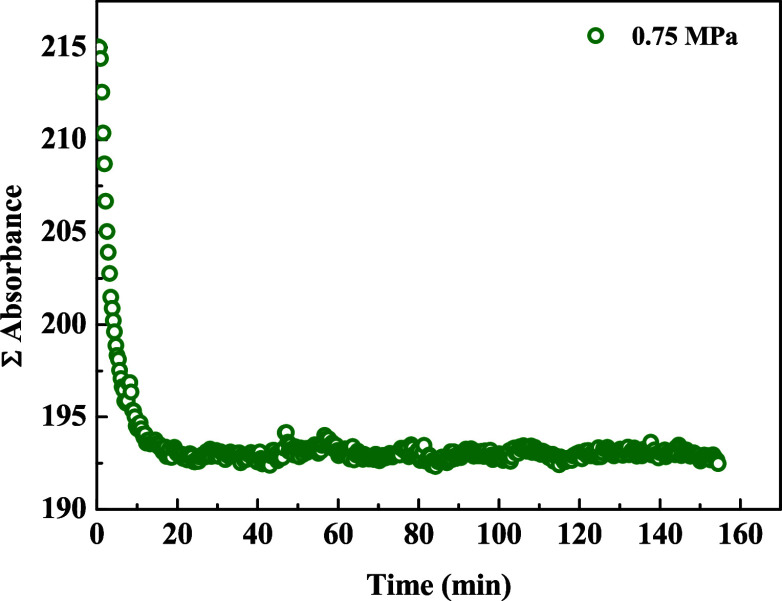
Variation of absorbance as a function of time during the CO_2_ dissolution process in water at 40 °C. The initial injection was performed at 1.01 MPa, with pressure decreasing until stabilization at 0.75 MPa.

Figure 3 shows the evolution of the sum of absorbances within this range, indirectly representing the incorporation of CO_2_ into water. The spectroscopic analysis was conducted in the near-infrared region, focusing on bands associated with the hydroxyl group (OH), particularly the first overtone of the stretching vibration (∼6500 cm^–1^)^16,17^ and the combination band of stretching and bending modes (∼7500 cm^–1^).^15,34,35^ These spectral regions are particularly sensitive to changes in the intermolecular interactions of water induced by the presence of dissolved CO_2_ and are crucial for real-time monitoring of the solubility process.

At a pressure of 0.87 MPa and temperature of 40 °C, NIR spectra were acquired in the range of 5500 to 9500 cm^–1^ (Figure 4), revealing progressive changes in absorption bands as the CO_2_ dissolution process advanced. Spectra were collected continuously from the same sample throughout the experiment. As the dissolution of CO_2_ progressed, a systematic decrease in the intensity of the bands associated with OH group vibrations were observed, particularly between 6500 and 7500 cm^–1^. This attenuation indicates alterations in water’s hydrogen bonding, possibly due to the reorganization of the hydrogen-bonding network as a result of interactions with dissolved CO_2_ molecules. Such behavior is consistent with the formation of solvation structures, directly reflected in the vibrational modes of the aqueous phase, an effect also documented in the literature.^14–17,36^

**Figure 4:**
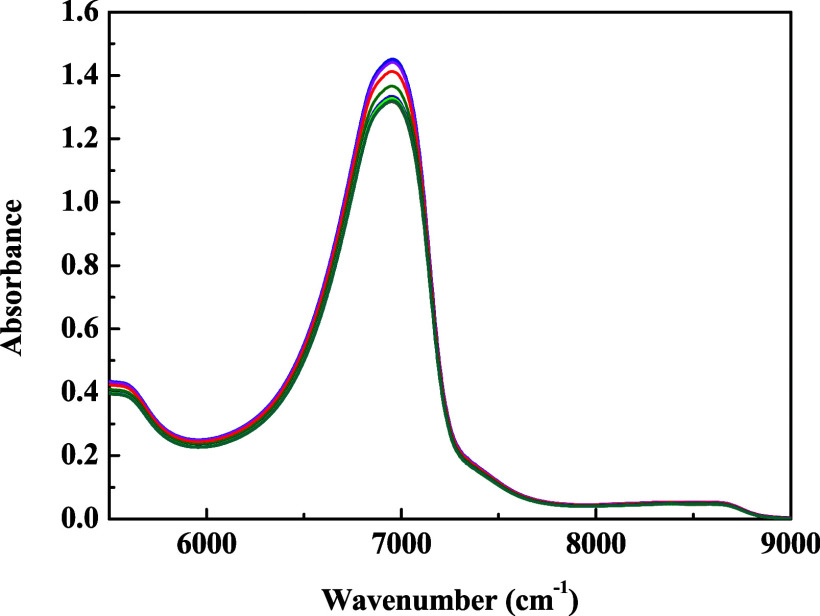
NIR absorption spectra (during CO_2_ dissolution in water) in the region from 5500 to 9500 cm^–1^, at 40 °C and 0.87 MPa, highlighting the absorbance intensity reduction. The spectra presented were collected by illuminating the same water sample as a function of the time.

Furthermore, the decreasing rate of spectral change over time indicated that the system was approaching equilibrium, marked by the stabilization of both spectral and physicochemical variables (pressure and temperature). The absence of new spectral bands suggested that, under the studied conditions, the dissolution of CO_2_ occurs predominantly as a physical solution. Nonetheless, it is important to note that ionic species such as bicarbonate and carbonate exhibit stretching vibrations of C=O near 1700 cm^–1^, which lie outside the analyzed NIR range. Therefore, while the present results indicate the predominance of physical dissolution, they do not fully exclude the possible formation of ionic species. Complementary measurements in the MIR region would be required to confirm their absence. Ahead, restricting the spectral region to the NIR, favoring the vibrational modes of the first overtone of water, allows the application of the method proposed here to any substances that may be dissolved in this liquid phase.

Accordingly, the observed spectral variations are primarily attributed to physical dissolution rather than chemical transformation, reinforcing the method’s specificity for monitoring gas–liquid equilibrium rather than secondary reactions.

Figure 5 presents the equilibrium spectra obtained at different pressures (0.25 to 1.52 MPa) at 40 °C. An increase in absorption intensity was observed with increasing pressure, especially between 5500 and 7500 cm^–1^. Ultrapure water, used as a reference, showed lower intensities, corroborating the impact of CO_2_ presence on the spectral properties of water. At higher pressures, the spectra became more intense and well-defined, suggesting a higher concentration of dissolved CO_2_, consistent with the principles of gas solubility.^5,12,16,35^

**Figure 5:**
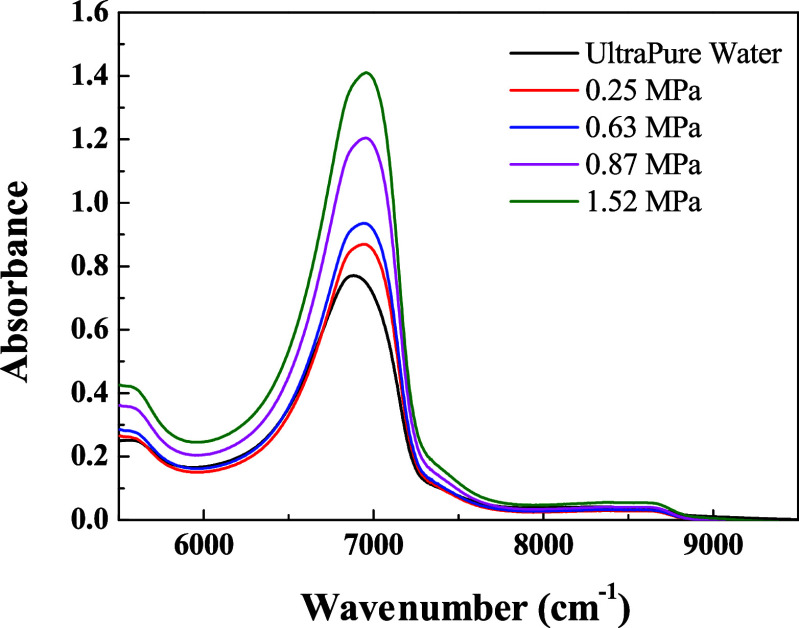
NIR absorbance spectra for CO_2_ dissolution in ultrapure water at 40 °C for 0.25 to 1.52 MPa equilibrium pressure values, collected in the transmission mode. Spectral changes in the 5500–9500 cm^–1^ range reflect the progressive saturation of CO_2_ under continuous stirring.

The influence of pressure was also noted: at higher pressure, the bands exhibited more pronounced shifts and intensity variations, which can be attributed to changes in the hydrogen bond network and the reorganization of water molecular conformations. These findings highlight the potential of NIR spectroscopy as a sensitive tool for investigating structural changes in multicomponent aqueous systems.^22^

Based on experimental data obtained from this work and values from the literature, a Peng–Robinson equation of state combined with the NRTL activity coefficient model was employed to estimate the solubility of CO_2_ in water under different temperature and pressure conditions. Figure 6 shows the parity plot between experimental values and those predicted by the thermodynamic model, with a mean absolute deviation of only 0.073%, highlighting its precision and confirming a good fit to the experimental data. These results validate the adequacy of the selected thermodynamic approach and reinforce the reliability of the methodology used in this study for solubility prediction under moderate to high-pressure and variable temperature conditions.

**Figure 6:**
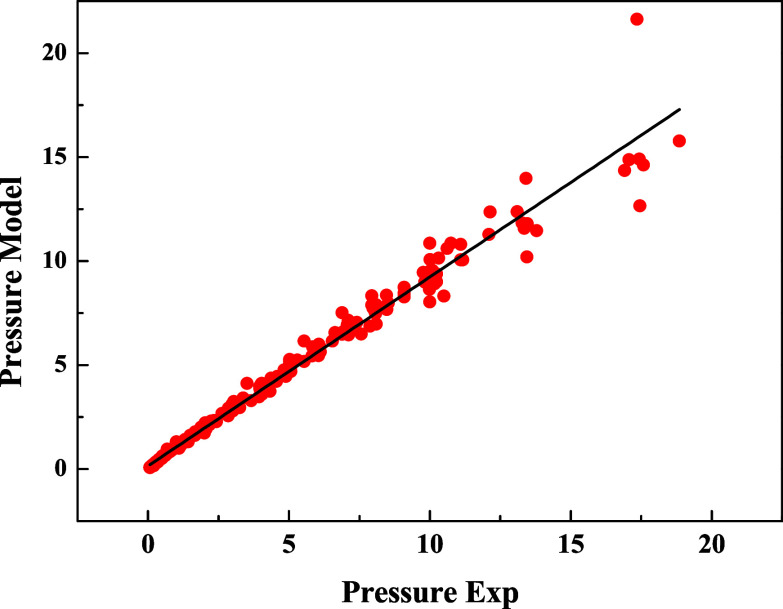
Parity plot for the CO_2_–water binary system: pressure calculated by the thermodynamic model (pressure model in MPa) versus experimental pressures from literature data (pressure exp in MPa), for temperatures ranging from 1 to 175 °C and CO_2_ concentrations from 0.0002 to 0.0296 mole fraction.

Table 2 lists the binary interaction parameters of the model, based on the equation of state and the activity coefficient model. The parameter *k_ij_* was kept zero, due to low solubility and pressure conditions under which specific interactions are reduced.

**Table 2:** Binary Interaction Parameters between CO_2_ (*i*) and Water (*j*) Corresponding to the Model Built with the Peng–Robinson Equation of State and the NRTL Model

system	*k*_*ij*_	τ_*ij*_	τ_*ji*_	objective function
CO_2_-water	0	–1.898	6.130	13975.299

Figure 7 shows the correlation between predicted and observed values for the concentration of dissolved CO_2_, using the PLS model applied to NIR spectra obtained under different temperature (40 to 60 °C) and pressure (0.15 to 2.35 MPa) conditions. The modeling process was conducted in two stages: calibration, using reference data, and validation, with an independent set of data not used in model construction.

**Figure 7:**
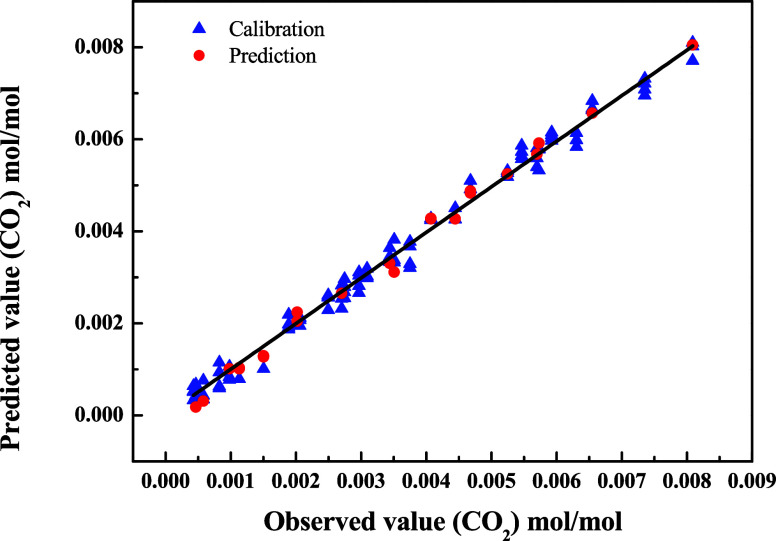
Predicted values versus observed values at different CO_2_ concentrations using the NIR spectra absorption data at different temperatures (between 40 and 60 °C) and pressure conditions (from 0.15 to 2.35 MPa). Calibration data (▲) and prediction data (**●**).

In the calibration stage, the PLS algorithm built a multivariate statistical model capable of relating spectral variables to actual CO_2_ concentration values. External validation was carried out with 809 spectra acquired under varied experimental conditions, demonstrating that the model maintained high predictive power even when applied to new data. The good distribution of points around the identity line (Figure 8) confirms the model’s accuracy in predicting the concentration of dissolved CO_2_.

**Figure 8:**
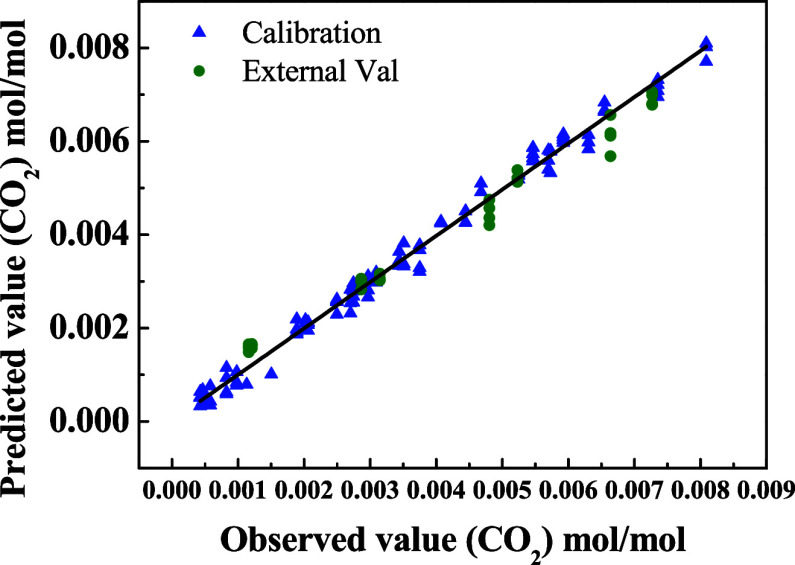
Correlation between predicted and observed values for CO_2_ concentration (mol/mol), using the PLS model applied to NIR spectra at different temperatures (between 45 and 55 °C) and pressure conditions (from 0.15 to 2.35 MPa). Calibration data (▲) and external validation data (**●**).

Table 3 presents the statistical parameters obtained, including root-mean-square errors (RMSE) and determination coefficients (*R* ^2^). The *R* ^2^ value greater than 0.99, as well as the proximity between calibration (RMSEC) and prediction (RMSEP) errors, and the mean external prediction error (9.91%), reinforce the robustness and reliability of the model for applications in spectral monitoring of CO_2_ solubility in water.

**Table 3:** Statistical Parameters of the PLS Model: CO_2_ Solubility in Water

								calibration		prediction	
system	*T* (°C)	factors	RMSECV	RMSEC	RMSEP	RMSEP_Ext_	Er._Ext_(%)	Er. (%)	*R* ^2^	Er.(%)	*R* ^2^
CO_2_-water	40–60	15	0.0002	0.0001	0.0001	0.0003	12.16	8.79	0.9903	9.91	0.9925

The selection of the number of latent components was based on the analysis of RMSECV, as shown in Figure 9. A sharp drop in RMSECV is observed up to approximately 8 components, followed by stabilization. Nevertheless, the final model was constructed with 15 components, as this point corresponds to the lowest absolute value of RMSECV and ensures greater coverage in explaining the variability of spectral data without compromising generalizations.

**Figure 9:**
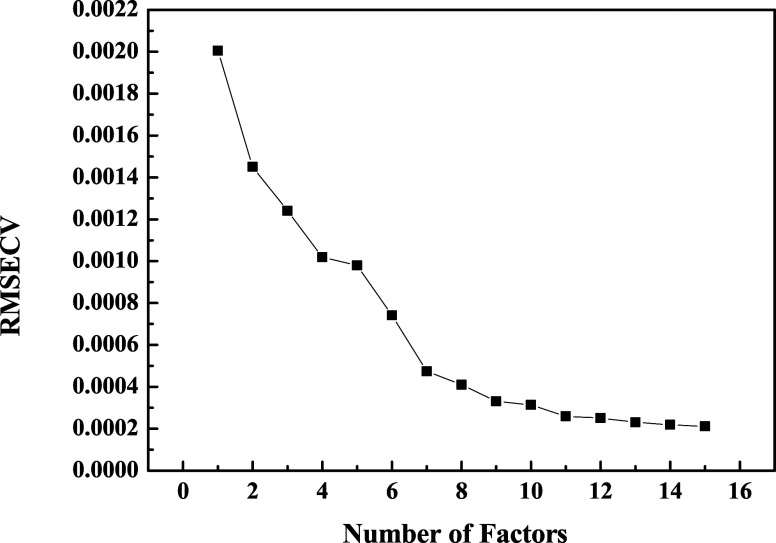
Root mean square error curve of cross-validation (RMSECV) as a function of the number of latent components of the PLS model.

Figure 10 shows the variation in the mole fraction of CO_2_ as a function of pressure at different temperatures (40 to 60 °C). The experimental and predicted results show excellent agreement, demonstrating the applicability of the developed approach for estimates under extended conditions, up to 175 °C. This broadens the applicability of the model used in this study to industrial processes. Importantly, the proposed model differs from conventional approaches by integrating NIR spectroscopy with thermodynamic modeling, enabling real-time estimation of CO_2_ solubility in aqueous systems. While classical models^1,4,5,36,37^ rely exclusively on equilibrium experimental data, our approach combines thermodynamic predictions with spectral information, to decrease uncertainties and extending industrial applicability.

**Figure 10:**
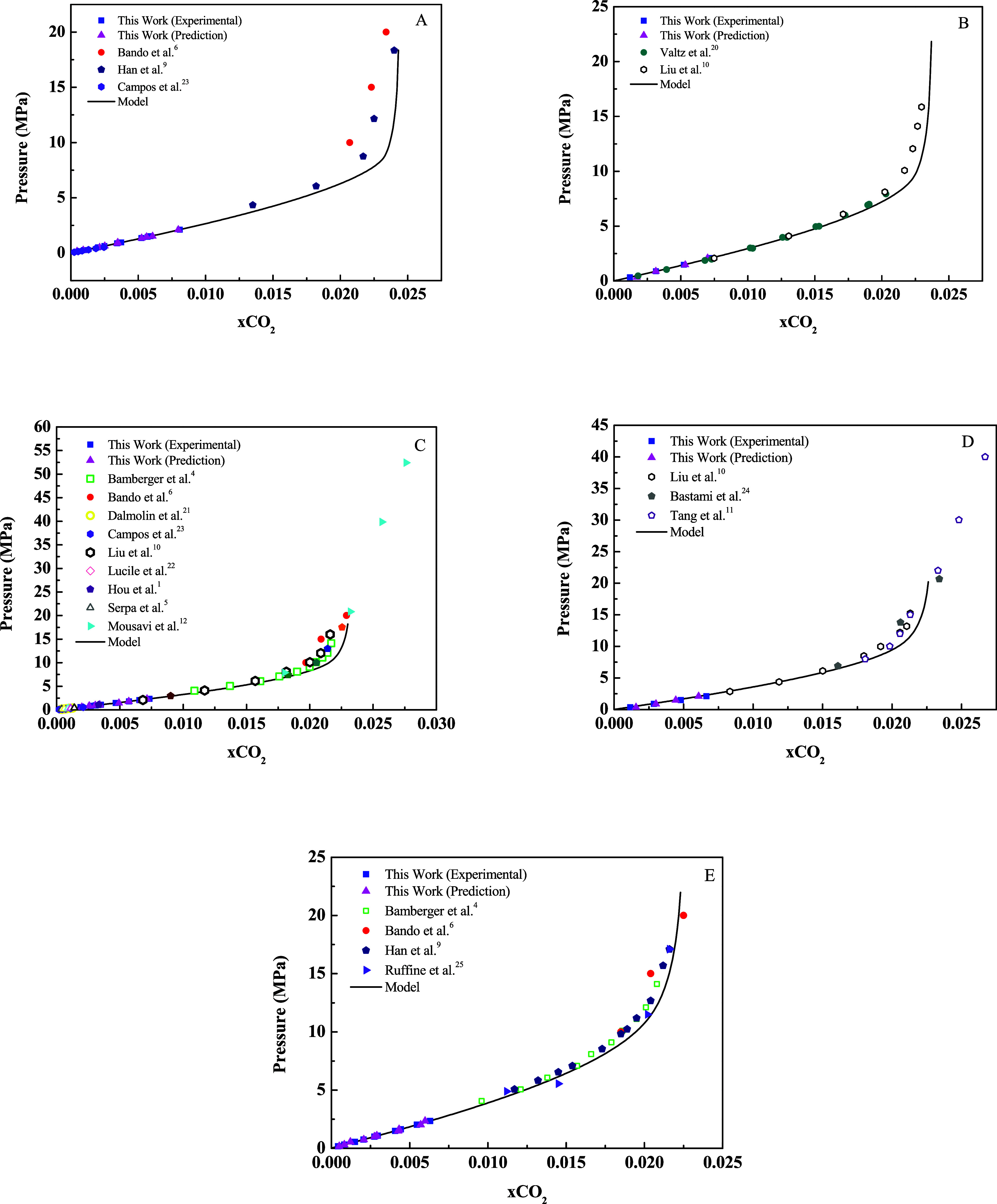
Mole fraction of CO_2_ (*x*CO_2_) in the aqueous phase as a function of pressure at different temperatures: (A) 40 °C, (B) 45 °C, (C) 50 °C, (D) 55 °C, and (E) 60 °C. Symbols represent experimental data and lines represent the thermodynamic model.

The data presented in Table 4 confirms the expected trend for the CO_2_ in water system: the solubility of CO_2_ increases with increasing pressure and decreases with increasing temperature. The table contains the experimental conditions of pressure and temperature, followed by its Real Concentration, obtained from thermodynamic modeling, and Predicted Concentration by the PLS regression model, as well as the absolute deviations between them (Absolute Error). The excellent agreement with the literature report reinforces the validity of the procedures adopted and the reliability of NIR spectroscopy as a robust analytical tool.

**Table 4:** Experimental Data for the Solubility of CO_2_ (1) in Water (2), Including the Real and Predicted Concentrations and Its Absolute Errors, at Different Temperature (40 to 60 °C) and Pressure (0.15 to 2.35 MPa) Conditions

temperature (°C)	pressure (MPa)	real concentration *x*_1_ × 10^2^	predicted concentration *x*_1_ × 10^2^	absolute error
40	0.15	0.0578	0.0461	0.00021
	0.25	0.0982	0.0921	0.00011
	0.51	0.2021	0.2142	0.00012
	0.63	0.2497	0.2512	0.00012
	0.87	0.3440	0.3432	0.00027
	0.95	0.3750	0.3773	0.00011
	1.34	0.5246	0.5254	0.00014
	1.46	0.5699	0.5618	0.00014
	1.52	0.5924	0.6062	0.00004
	2.11	0.8088	0.7972	0.00012
45	0.34	0.1211	0.1722	0.00041
	0.88	0.3147	0.3139	0.00009
	1.48	0.5232	0.5331	0.00008
	2.09	0.7270	0.6998	0.00037
50	0.15	0.0463	0.0485	0.00018
	0.35	0.1129	0.0963	0.00017
	0.58	0.1889	0.1988	0.00011
	0.83	0.2704	0.2584	0.00018
	0.95	0.3091	0.3055	0.00026
	1.08	0.3508	0.3409	0.00009
	1.45	0.4678	0.4932	0.00025
	1.79	0.5730	0.5706	0.00018
	2.06	0.6549	0.6677	0.00013
	2.33	0.7352	0.7144	0.00021
55	0.36	0.1163	0.1570	0.00041
	0.88	0.2866	0.3008	0.00013
	1.49	0.4804	0.4441	0.00034
	2.09	0.6639	0.6081	0.00051
60	0.17	0.0429	0.0504	0.00012
	0.31	0.0827	0.0829	0.00022
	0.55	0.1502	0.1217	0.00028
	0.75	0.2059	0.2066	0.00006
	0.99	0.2745	0.2749	0.00013
	1.08	0.2967	0.2907	0.00017
	1.49	0.4073	0.4269	0.00020
	1.63	0.4444	0.4327	0.00015
	2.02	0.5464	0.5704	0.00024
	2.35	0.6307	0.5985	0.00032

The comparative analysis reveals good agreement between the modeled concentrations and data reported in the literature for similar conditions of analyses,^4,6,10,19^ which reinforces the validity of the adopted procedures. Furthermore, the low absolute errors observed between the predicted and thermodynamically calculated values highlight the robustness of the proposed chemometric model and demonstrate the reliability of NIR spectroscopy as an analytical tool for monitoring vapor–liquid equilibrium in aqueous systems containing CO_2_.

## 4 Conclusions

This study proposed and validated a methodology for monitoring the solubility of CO_2_ in water using NIR spectroscopy, thermodynamic modeling, and chemometrics tools. These integrated applications enabled not only real-time monitoring of the CO_2_ dissolution process but also precise prediction of the dissolved gas concentration under different temperature and pressure conditions.

Spectral analysis demonstrated that the NIR spectroscopy technique is sensitive to structural changes in water induced by the dissolution of CO_2_, allowing for rapid, noninvasive detection of molecular variations associated with vapor–liquid phase equilibrium without the need for phase separation. Thermodynamic modeling, based on the γ-ϕ approach, showed excellent performance, with mean deviations below 0.1% in equilibrium pressure predictions. In parallel, the chemometric model based on partial least-squares regression (PLS) proved to be highly predictive, with coefficients of determination (*R* ^2^ > 0.99) and low error values, validating its high capacity to estimate dissolved CO_2_ concentrations from spectra obtained in real time.

The results obtained demonstrate the feasibility of the proposed approach as a robust and efficient alternative for the study of multicomponent systems, especially in industrial contexts that require strict process control, such as in the oil, gas, environmental, and process engineering sectors. This integration of NIR spectroscopy, physicochemical modeling, and multivariate analysis opens new possibilities for the development of smart sensors and process monitoring under extreme operational conditions in the industry.

Although the present study was restricted to the CO_2_–H_2_O system, the NIR-based methodology can be extended to hydrocarbon-CO_2_ mixtures and synthetic saline solutions, since the analyses are centered on the region of the first water overtone, of the NIR spectrum, provided that calibration adjustments and adaptation of optical probes are performed. This possibility represents a relevant perspective for applications in EOR, geological carbon sequestration, and the monitoring of multicomponent industrial processes. Furthermore, the methodology can be applied to aqueous saline systems (NaCl, CaCl_2_, MgCl_2_) and systems containing alkanolamines, which are highly relevant for carbon capture. These developments are already being considered as the focus of future work, aiming to demonstrate the practical applicability of the technique in more complex industrial systems.

## Supplementary Material



## Notes

Experimental data sets on the solubility of CO_2_ in water under different temperatures and pressures, a complete compilation of literature data employed for the parametrization of thermodynamic models, references to experimental studies, and descriptions of the measurement conditions and data ranges (temperatures from 1 to 175 °C and pressures up to 52.4 MPa) (PDF)
