# Supplementary material for: Determination of CO2 Solubility in Water by NIR Spectroscopy under Different Temperature and Pressure Conditions
Source: ACS Omega. 2025 Nov 28;10(48):58714–23. doi: 10.1021/acsomega.5c07060 (PMC12771439; doi:10.1021/acsomega.5c07060)
Supplement: Supplementary file 1 [file ao5c07060_si_001.pdf]

Determination of CO_2 solubility in water by NIR spectroscopy under different temperature and pressure conditions

Lorena Armando da Silveira[a], Lorena Mariah Oliveira Lima[a], Thiago Rodrigues da Cunha[a], Fabiane Santos Serpa[a], Ranyere Lucena Souza[a], Rosane Alves Fontes[c], Giselle Maria Lopes Leite da Silva[c], Mônica Teixeira da Silva[c], Jussara de Mello Silva[c], Tiago Cavalcante Freitas[c], Luiz Alexandre Sacorague[c], Claudio Dariva[a], Marcos Lúcio Corazza[b], Elton Franceschi[a]

[a]Tiradentes University, Center for Studies on Colloidal Systems, Institute of Technology and Research, Av. Murilo Dantas, 300, Aracaju, SE, BR 49032-490

[b]Federal University of Parana, Chemical Engineering Department, Rua Coronel Francisco Heráclito dos Santos, 210, Curitiba, PR, BR 81531-990

[c]Petróleo Brasileiro SA, Research Center Leopoldo Américo Miguez de Mello, Av. Horácio Macedo, 950, Ilha do Fundão, Rio de Janeiro, RJ, BR 20031-912

1. Experimental Database for the CO_2-H_2O System

Table A.1 presents the experimental data compiled from literature and used for the parametrization of the thermodynamic model for the CO_2-H_2O system. The database comprises 510 measurements of CO_2 solubility in the aqueous phase, covering a temperature range from 1 to 175 °C and pressures up to 52.4 MPa.

Table A.1. *Experimental data on the solubility of CO_2 in water at different temperatures and pressures, expressed as mole fractions of CO_2 and H_2O in the aqueous phase.*

Temperature	Pressure	Mole Fraction		Reference
°C	MPa	x (CO_2)	x (H_2O)	
1	0.076	0.00099	0.99901	Anderson (2002)
1	0.251	0.00321	0.99679	Anderson (2002)
1	0.472	0.00606	0.99394	Anderson (2002)
1	0.775	0.00960	0.99040	Anderson (2002)
1	1.056	0.01276	0.98724	Anderson (2002)
1	1.362	0.01601	0.98399	Anderson (2002)
1	0.190	0.00262	0.99738	Chapoy et al. (2004)
1	1.201	0.01420	0.98580	Chapoy et al. (2004)
3	0.079	0.00097	0.99903	Anderson (2002)
3	0.262	0.00312	0.99688	Anderson (2002)
3	0.495	0.00586	0.99414	Anderson (2002)
3	0.809	0.00930	0.99070	Anderson (2002)
3	1.100	0.01240	0.98760	Anderson (2002)
3	1.420	0.01550	0.98450	Anderson (2002)
3	1.327	0.01420	0.98580	Chapoy et al. (2004)
5	2.000	0.01830	0.98170	Servio & Englezos (2001)
5	0.083	0.00094	0.99906	Anderson (2002)
5	0.274	0.00303	0.99697	Anderson (2002)
5	0.516	0.00570	0.99430	Anderson (2002)
5	0.842	0.00902	0.99098	Anderson (2002)
5	1.146	0.01200	0.98800	Anderson (2002)
5	1.479	0.01498	0.98502	Anderson (2002)
5	1.858	0.01772	0.98228	Anderson (2002)
5	0.501	0.00585	0.99415	Valtz et al. (2004)
5	0.755	0.00852	0.99148	Valtz et al. (2004)
5	1.016	0.01111	0.98889	Valtz et al. (2004)
5	1.322	0.01403	0.98597	Valtz et al. (2004)
5	1.674	0.01747	0.98253	Valtz et al. (2004)
5	2.031	0.02015	0.97985	Valtz et al. (2004)
5	0.228	0.00262	0.99738	Chapoy et al. (2004)

5	1.426	0.01420	0.98580	Chapoy et al. (2004)
7	2.000	0.01700	0.98300	Servio & Englezos (2001)
7	0.086	0.00091	0.99909	Anderson (2002)
7	0.285	0.00294	0.99706	Anderson (2002)
7	0.538	0.00552	0.99448	Anderson (2002)
7	0.876	0.00873	0.99127	Anderson (2002)
7	1.194	0.01160	0.98840	Anderson (2002)
7	1.538	0.01447	0.98553	Anderson (2002)
7	1.924	0.01714	0.98286	Anderson (2002)
9	2.000	0.01560	0.98440	Servio & Englezos (2001)
9	0.090	0.00088	0.99912	Anderson (2002)
9	0.297	0.00286	0.99714	Anderson (2002)
9	0.560	0.00535	0.99465	Anderson (2002)
9	0.906	0.00849	0.99151	Anderson (2002)
9	1.237	0.01125	0.98875	Anderson (2002)
9	1.589	0.01405	0.98595	Anderson (2002)
9	1.982	0.01666	0.98334	Anderson (2002)
11	0.094	0.00085	0.99915	Anderson (2002)
11	0.309	0.00277	0.99723	Anderson (2002)
11	0.580	0.00520	0.99480	Anderson (2002)
11	0.940	0.00822	0.99178	Anderson (2002)
11	1.289	0.01082	0.98918	Anderson (2002)
11	1.641	0.01363	0.98637	Anderson (2002)
11	2.051	0.01606	0.98394	Anderson (2002)
11	0.287	0.00262	0.99738	Chapoy et al. (2004)
11	3.938	0.02688	0.97312	Chapoy et al. (2004)
13	0.097	0.00083	0.99917	Anderson (2002)
13	0.320	0.00269	0.99731	Anderson (2002)
13	0.600	0.00505	0.99495	Anderson (2002)
13	0.971	0.00797	0.99203	Anderson (2002)
13	1.336	0.01045	0.98955	Anderson (2002)
13	1.996	0.01318	0.98682	Anderson (2002)
13	2.113	0.01554	0.98446	Anderson (2002)
13	0.200	0.00130	0.99870	Muromachi et al. (2015)
13	0.500	0.00410	0.99590	Muromachi et al. (2015)
13	1.000	0.00820	0.99180	Muromachi et al. (2015)
15	0.101	0.00080	0.99920	Anderson (2002)
15	0.332	0.00260	0.99740	Anderson (2002)
15	0.620	0.00490	0.99510	Anderson (2002)
15	0.998	0.00777	0.99223	Anderson (2002)
15	1.376	0.01015	0.98985	Anderson (2002)
15	1.748	0.01278	0.98722	Anderson (2002)
15	2.179	0.01500	0.98500	Anderson (2002)
15	0.329	0.00262	0.99738	Chapoy et al. (2004)

15	0.115	0.00093	0.99907	Dalmolin et al. (2006)
15	0.125	0.00101	0.99899	Dalmolin et al. (2006)
15	0.134	0.00112	0.99888	Dalmolin et al. (2006)
15	0.171	0.00143	0.99857	Dalmolin et al. (2006)
15	0.196	0.00160	0.99840	Dalmolin et al. (2006)
15	0.210	0.00164	0.99836	Dalmolin et al. (2006)
15	0.264	0.00215	0.99785	Dalmolin et al. (2006)
15	0.269	0.00212	0.99788	Dalmolin et al. (2006)
15	0.272	0.00213	0.99787	Dalmolin et al. (2006)
15	0.345	0.00270	0.99730	Dalmolin et al. (2006)
15	0.348	0.00273	0.99727	Dalmolin et al. (2006)
15	0.365	0.00297	0.99703	Dalmolin et al. (2006)
15	0.409	0.00325	0.99675	Dalmolin et al. (2006)
15	0.466	0.00365	0.99635	Dalmolin et al. (2006)
19	0.200	0.00110	0.99890	Muromachi et al. (2015)
19	0.500	0.00360	0.99640	Muromachi et al. (2015)
19	1.000	0.00710	0.99290	Muromachi et al. (2015)
19	2.000	0.01340	0.98660	Muromachi et al. (2015)
19	3.000	0.01840	0.98160	Muromachi et al. (2015)
19	4.000	0.02340	0.97660	Muromachi et al. (2015)
20	0.385	0.00262	0.99738	Chapoy et al. (2004)
20	2.349	0.01420	0.98580	Chapoy et al. (2004)
20	0.092	0.00063	0.99937	Dalmolin et al. (2006)
20	0.103	0.00071	0.99929	Dalmolin et al. (2006)
20	0.170	0.00115	0.99885	Dalmolin et al. (2006)
20	0.188	0.00133	0.99867	Dalmolin et al. (2006)
20	0.234	0.00157	0.99843	Dalmolin et al. (2006)
20	0.269	0.00182	0.99818	Dalmolin et al. (2006)
20	0.294	0.00202	0.99798	Dalmolin et al. (2006)
20	0.353	0.00242	0.99758	Dalmolin et al. (2006)
20	0.361	0.00249	0.99751	Dalmolin et al. (2006)
25	0.504	0.00314	0.99686	Valtz et al. (2004)
25	1.007	0.00614	0.99386	Valtz et al. (2004)
25	1.496	0.00887	0.99113	Valtz et al. (2004)
25	2.843	0.01356	0.98644	Valtz et al. (2004)
25	3.941	0.01772	0.98228	Valtz et al. (2004)
25	4.492	0.02090	0.97910	Valtz et al. (2004)
25	5.524	0.02323	0.97677	Valtz et al. (2004)
25	0.101	0.00059	0.99941	Dalmolin et al. (2006)
25	0.188	0.00107	0.99893	Dalmolin et al. (2006)
25	0.261	0.00155	0.99845	Dalmolin et al. (2006)
25	0.244	0.00149	0.99851	Dalmolin et al. (2006)
25	0.304	0.00185	0.99815	Dalmolin et al. (2006)
25	0.368	0.00220	0.99780	Dalmolin et al. (2006)

25	0.401	0.00244	0.99756	Dalmolin et al. (2006)
25	0.466	0.00284	0.99716	Dalmolin et al. (2006)
25	0.263	0.00170	0.99830	Dell'Era et al. (2010)
25	0.435	0.00270	0.99730	Dell'Era et al. (2010)
25	0.531	0.00320	0.99680	Dell'Era et al. (2010)
25	0.59	0.00360	0.99640	Dell'Era et al. (2010)
25	0.664	0.00400	0.99600	Dell'Era et al. (2010)
25	0.707	0.00430	0.99570	Dell'Era et al. (2010)
25	0.747	0.00460	0.99540	Dell'Era et al. (2010)
25	0.610	0.00362	0.99638	Lucile et al. (2012)
25	0.680	0.00534	0.99466	Lucile et al. (2012)
25	1.970	0.00985	0.99015	Lucile et al. (2012)
25	3.010	0.01470	0.98530	Lucile et al. (2012)
25	4.040	0.01880	0.98120	Lucile et al. (2012)
25	4.730	0.02220	0.97780	Lucile et al. (2012)
25	1.666	0.00887	0.99113	Hou et al. (2013)
25	3.258	0.01540	0.98460	Hou et al. (2013)
25	6.391	0.02484	0.97516	Hou et al. (2013)
25	10.183	0.02630	0.97370	Hou et al. (2013)
25	13.434	0.02782	0.97218	Hou et al. (2013)
25	17.551	0.02944	0.97056	Hou et al. (2013)
25	0.154	0.00090	0.99910	Serpa et al. (2013)
25	0.253	0.00150	0.99850	Serpa et al. (2013)
25	0.326	0.00190	0.99810	Serpa et al. (2013)
25	0.378	0.00230	0.99770	Serpa et al. (2013)
25	0.200	0.00090	0.99910	Muromachi et al. (2015)
25	0.500	0.00280	0.99720	Muromachi et al. (2015)
25	1.000	0.00640	0.99360	Muromachi et al. (2015)
25	3.000	0.01630	0.98370	Muromachi et al. (2015)
25	2.000	0.01140	0.98860	Muromachi et al. (2015)
25	4.000	0.02000	0.98000	Muromachi et al. (2015)
28	2.071	0.01099	0.98901	Ahmadi & Chapoy. (2018)
28	4.782	0.02032	0.97968	Ahmadi & Chapoy. (2018)
28	7.837	0.02335	0.97665	Ahmadi & Chapoy. (2018)
28	19.445	0.02696	0.97304	Ahmadi & Chapoy. (2018)
28	34.753	0.02960	0.97040	Ahmadi & Chapoy. (2018)
30	10.000	0.02380	0.97620	Bando et al. (2003)
30	15.000	0.02480	0.97520	Bando et al. (2003)
30	20.000	0.02570	0.97430	Bando et al. (2003)
30	0.064	0.00031	0.99969	Campos et al. (2009)
30	0.124	0.00067	0.99933	Campos et al. (2009)
30	0.188	0.00104	0.99896	Campos et al. (2009)
30	0.281	0.00161	0.99839	Campos et al. (2009)
30	0.392	0.00229	0.99771	Campos et al. (2009)

30	0.511	0.00307	0.99693	Campos et al. (2009)
31	1.013	0.00626	0.99374	Zhang et al. (2005)
31	2.026	0.00960	0.99040	Zhang et al. (2005)
31	3.039	0.01330	0.98670	Zhang et al. (2005)
31	4.558	0.01800	0.98200	Zhang et al. (2005)
35	0.579	0.00276	0.99724	Valtz et al. (2004)
35	1.889	0.00856	0.99144	Valtz et al. (2004)
35	2.950	0.01212	0.98788	Valtz et al. (2004)
35	3.029	0.01260	0.98740	Valtz et al. (2004)
35	4.005	0.01563	0.98437	Valtz et al. (2004)
35	4.985	0.01837	0.98163	Valtz et al. (2004)
35	5.949	0.02033	0.97967	Valtz et al. (2004)
35	6.077	0.02066	0.97934	Valtz et al. (2004)
35	6.972	0.02230	0.97770	Valtz et al. (2004)
35	6.986	0.02152	0.97848	Valtz et al. (2004)
35	7.029	0.02221	0.97779	Valtz et al. (2004)
35	7.963	0.02304	0.97696	Valtz et al. (2004)
35	0.109	0.00052	0.99948	Dalmolin et al. (2006)
35	0.136	0.00064	0.99936	Dalmolin et al. (2006)
35	0.228	0.00108	0.99892	Dalmolin et al. (2006)
35	0.277	0.00132	0.99868	Dalmolin et al. (2006)
35	0.298	0.00142	0.99858	Dalmolin et al. (2006)
35	0.345	0.00163	0.99837	Dalmolin et al. (2006)
35	0.361	0.00171	0.99829	Dalmolin et al. (2006)
35	0.410	0.00194	0.99806	Dalmolin et al. (2006)
35	0.442	0.00208	0.99792	Dalmolin et al. (2006)
35	0.473	0.00226	0.99774	Dalmolin et al. (2006)
35	2.100	0.00933	0.99067	Liu et al. (2011)
35	4.090	0.01574	0.98426	Liu et al. (2011)
35	6.080	0.02035	0.97965	Liu et al. (2011)
35	8.090	0.02240	0.97760	Liu et al. (2011)
35	10.080	0.02314	0.97686	Liu et al. (2011)
35	12.050	0.02375	0.97625	Liu et al. (2011)
35	14.010	0.02454	0.97546	Liu et al. (2011)
35	15.830	0.02494	0.97506	Liu et al. (2011)
35	7.981	0.02106	0.97894	Tang et al. (2015)
35	9.993	0.02198	0.97802	Tang et al. (2015)
35	12.005	0.02289	0.97711	Tang et al. (2015)
35	15.007	0.02370	0.97630	Tang et al. (2015)
35	21.999	0.02522	0.97478	Tang et al. (2015)
35	30.014	0.02662	0.97338	Tang et al. (2015)
35	40.007	0.02793	0.97207	Tang et al. (2015)
35	1.292	0.00601	0.99399	Ahmadi & Chapoy. (2018)
35	1.310	0.00601	0.99399	Ahmadi & Chapoy. (2018)

35	2.243	0.00974	0.99026	Ahmadi & Chapoy. (2018)
35	3.380	0.01384	0.98616	Ahmadi & Chapoy. (2018)
35	4.594	0.01704	0.98296	Ahmadi & Chapoy. (2018)
35	5.543	0.01931	0.98069	Ahmadi & Chapoy. (2018)
35	7.143	0.02228	0.97772	Ahmadi & Chapoy. (2018)
35	12.231	0.02414	0.97586	Ahmadi & Chapoy. (2018)
35	19.236	0.02589	0.97411	Ahmadi & Chapoy. (2018)
40	10.000	0.02070	0.97930	Bando et al. (2003)
40	15.000	0.02230	0.97770	Bando et al. (2003)
40	20.000	0.02340	0.97660	Bando et al. (2003)
40	1.070	0.00496	0.99504	Zhang et al. (2005)
40	2.030	0.00846	0.99154	Zhang et al. (2005)
40	4.590	0.01560	0.98440	Zhang et al. (2005)
40	3.040	0.01117	0.98883	Zhang et al. (2005)
40	4.330	0.01350	0.98650	Han et al. (2009)
40	6.050	0.01820	0.98180	Han et al. (2009)
40	8.750	0.02170	0.97830	Han et al. (2009)
40	12.150	0.02250	0.97750	Han et al. (2009)
40	18.340	0.02400	0.97600	Han et al. (2009)
40	0.068	0.00025	0.99975	Campos et al. (2009)
40	0.129	0.00054	0.99946	Campos et al. (2009)
40	0.193	0.00084	0.99916	Campos et al. (2009)
40	0.288	0.00131	0.99869	Campos et al. (2009)
40	0.401	0.00186	0.99814	Campos et al. (2009)
40	0.524	0.00246	0.99754	Campos et al. (2009)
45	0.465	0.00182	0.99818	Valtz et al. (2004)
45	1.045	0.00394	0.99606	Valtz et al. (2004)
45	1.863	0.00680	0.99320	Valtz et al. (2004)
45	1.984	0.00730	0.99270	Valtz et al. (2004)
45	2.970	0.01036	0.98964	Valtz et al. (2004)
45	3.001	0.01018	0.98982	Valtz et al. (2004)
45	3.977	0.01260	0.98740	Valtz et al. (2004)
45	3.969	0.01293	0.98707	Valtz et al. (2004)
45	4.952	0.01508	0.98492	Valtz et al. (2004)
45	4.982	0.01532	0.98468	Valtz et al. (2004)
45	5.978	0.01720	0.98280	Valtz et al. (2004)
45	5.992	0.01726	0.98274	Valtz et al. (2004)
45	6.923	0.01895	0.98105	Valtz et al. (2004)
45	6.984	0.01905	0.98095	Valtz et al. (2004)
45	7.933	0.02031	0.97969	Valtz et al. (2004)
45	2.080	0.00749	0.99251	Liu et al. (2011)
45	4.100	0.01305	0.98695	Liu et al. (2011)
45	6.090	0.01711	0.98289	Liu et al. (2011)
45	8.110	0.02022	0.97978	Liu et al. (2011)

45	10.080	0.02170	0.97830	Liu et al. (2011)
45	12.060	0.02231	0.97769	Liu et al. (2011)
45	14.110	0.02266	0.97734	Liu et al. (2011)
45	15.860	0.02296	0.97704	Liu et al. (2011)
50	4.050	0.01090	0.98910	Bamberger et al. (2000)
50	5.060	0.01370	0.98630	Bamberger et al. (2000)
50	6.060	0.01610	0.98390	Bamberger et al. (2000)
50	7.080	0.01760	0.98240	Bamberger et al. (2000)
50	8.080	0.01900	0.98100	Bamberger et al. (2000)
50	9.090	0.02000	0.98000	Bamberger et al. (2000)
50	10.090	0.02050	0.97950	Bamberger et al. (2000)
50	11.100	0.02100	0.97900	Bamberger et al. (2000)
50	12.100	0.02140	0.97860	Bamberger et al. (2000)
50	14.110	0.02170	0.97830	Bamberger et al. (2000)
50	10.000	0.01970	0.98030	Bando et al. (2003)
50	15.000	0.02090	0.97910	Bando et al. (2003)
50	20.000	0.02290	0.97710	Bando et al. (2003)
50	0.114	0.00038	0.99962	Dalmolin. I. (2006)
50	0.133	0.00045	0.99955	Dalmolin. I. (2006)
50	0.176	0.00059	0.99941	Dalmolin. I. (2006)
50	0.214	0.00073	0.99927	Dalmolin. I. (2006)
50	0.246	0.00083	0.99917	Dalmolin. I. (2006)
50	0.304	0.00103	0.99897	Dalmolin. I. (2006)
50	0.306	0.00105	0.99895	Dalmolin. I. (2006)
50	0.365	0.00128	0.99872	Dalmolin. I. (2006)
50	0.075	0.00020	0.99980	Campos et al. (2009)
50	0.136	0.00045	0.99955	Campos et al. (2009)
50	0.199	0.00071	0.99929	Campos et al. (2009)
50	0.297	0.00111	0.99889	Campos et al. (2009)
50	0.535	0.00210	0.99790	Campos et al. (2009)
50	2.100	0.00682	0.99318	Liu et al. (2011)
50	4.110	0.01170	0.98830	Liu et al. (2011)
50	6.120	0.01570	0.98430	Liu et al. (2011)
50	8.100	0.01815	0.98185	Liu et al. (2011)
50	10.100	0.02001	0.97999	Liu et al. (2011)
50	12.040	0.02087	0.97913	Liu et al. (2011)
50	15.990	0.02161	0.97839	Liu et al. (2011)
50	0.600	0.00214	0.99786	Lucile et al. (2012)
50	1.050	0.00331	0.99669	Lucile et al. (2012)
50	2.060	0.00616	0.99384	Lucile et al. (2012)
50	2.980	0.00895	0.99105	Lucile et al. (2012)
50	4.090	0.01130	0.98870	Lucile et al. (2012)
50	4.120	0.01160	0.98840	Lucile et al. (2012)
50	5.020	0.01390	0.98610	Lucile et al. (2012)

50	1.089	0.00333	0.99667	Hou et al. (2013)
50	2.980	0.00901	0.99099	Hou et al. (2013)
50	7.406	0.01830	0.98170	Hou et al. (2013)
50	10.021	0.02054	0.97946	Hou et al. (2013)
50	12.973	0.02141	0.97859	Hou et al. (2013)
50	17.533	0.02255	0.97745	Hou et al. (2013)
50	0.125	0.00040	0.99960	Serpa et al. (2013)
50	0.215	0.00060	0.99940	Serpa et al. (2013)
50	0.289	0.00090	0.99910	Serpa et al. (2013)
50	0.349	0.00110	0.99890	Serpa et al. (2013)
50	0.408	0.00140	0.99860	Serpa et al. (2013)
50	7.876	0.01800	0.98200	Mousavi et al. (2024)
50	20.814	0.02320	0.97680	Mousavi et al. (2024)
50	39.855	0.02570	0.97430	Mousavi et al. (2024)
50	52.414	0.02760	0.97240	Mousavi et al. (2024)
55	2.860	0.00833	0.99167	Liu et al. (2011)
55	4.370	0.01187	0.98813	Liu et al. (2011)
55	6.110	0.01501	0.98499	Liu et al. (2011)
55	8.480	0.01797	0.98203	Liu et al. (2011)
55	9.990	0.01918	0.98082	Liu et al. (2011)
55	12.200	0.02057	0.97943	Liu et al. (2011)
55	13.190	0.02105	0.97895	Liu et al. (2011)
55	15.230	0.02131	0.97869	Liu et al. (2011)
55	6.890	0.01610	0.98390	Bastami et al. (2014)
55	13.790	0.02060	0.97940	Bastami et al. (2014)
55	20.680	0.02340	0.97660	Bastami et al. (2014)
55	7.981	0.01807	0.98193	Tang et al. (2015)
55	9.993	0.01983	0.98017	Tang et al. (2015)
55	12.005	0.02057	0.97943	Tang et al. (2015)
55	15.007	0.02131	0.97869	Tang et al. (2015)
55	21.999	0.02331	0.97669	Tang et al. (2015)
55	30.014	0.02481	0.97519	Tang et al. (2015)
55	40.007	0.02669	0.97331	Tang et al. (2015)
60	4.050	0.00960	0.99040	Bamberger et al. (2000)
60	5.060	0.01210	0.98790	Bamberger et al. (2000)
60	6.060	0.01380	0.98620	Bamberger et al. (2000)
60	7.080	0.01570	0.98430	Bamberger et al. (2000)
60	8.080	0.01660	0.98340	Bamberger et al. (2000)
60	9.090	0.01790	0.98210	Bamberger et al. (2000)
60	10.090	0.01860	0.98140	Bamberger et al. (2000)
60	11.100	0.01950	0.98050	Bamberger et al. (2000)
60	12.100	0.02010	0.97990	Bamberger et al. (2000)
60	14.110	0.02080	0.97920	Bamberger et al. (2000)
60	10.000	0.01850	0.98150	Bando et al. (2003)

60	15.000	0.02040	0.97960	Bando et al. (2003)
60	20.000	0.02250	0.97750	Bando et al. (2003)
60	5.070	0.01170	0.98830	Han et al. (2009)
60	5.820	0.01320	0.98680	Han et al. (2009)
60	6.540	0.01450	0.98550	Han et al. (2009)
60	7.070	0.01540	0.98460	Han et al. (2009)
60	8.530	0.01730	0.98270	Han et al. (2009)
60	9.820	0.01850	0.98150	Han et al. (2009)
60	10.240	0.01890	0.98110	Han et al. (2009)
60	11.170	0.01950	0.98050	Han et al. (2009)
60	12.670	0.02040	0.97960	Han et al. (2009)
60	15.690	0.02120	0.97880	Han et al. (2009)
60	17.100	0.02160	0.97840	Han et al. (2009)
60	4.890	0.01120	0.98880	Ruffine. L. (2010)
60	5.5400	0.01450	0.98550	Ruffine. L. (2010)
60	11.500	0.02020	0.97980	Ruffine. L. (2010)
70	5.300	0.01130	0.98870	Han et al. (2009)
70	5.840	0.01240	0.98760	Han et al. (2009)
70	6.640	0.01350	0.98650	Han et al. (2009)
70	7.110	0.01440	0.98560	Han et al. (2009)
70	7.940	0.01540	0.98460	Han et al. (2009)
70	8.470	0.01600	0.98400	Han et al. (2009)
70	10.310	0.01790	0.98210	Han et al. (2009)
70	10.620	0.01830	0.98170	Han et al. (2009)
70	10.750	0.01850	0.98150	Han et al. (2009)
70	12.140	0.01950	0.98050	Han et al. (2009)
70	14.140	0.02050	0.97950	Han et al. (2009)
70	17.260	0.02150	0.97850	Han et al. (2009)
75	0.540	0.00126	0.99874	Lucile et al. (2012)
75	1.000	0.00241	0.99759	Lucile et al. (2012)
75	2.030	0.00467	0.99533	Lucile et al. (2012)
75	3.100	0.00688	0.99312	Lucile et al. (2012)
75	4.030	0.00859	0.99141	Lucile et al. (2012)
75	5.140	0.01060	0.98940	Lucile et al. (2012)
75	1.101	0.00222	0.99778	Hou et al. (2013)
75	2.921	0.00608	0.99392	Hou et al. (2013)
75	7.123	0.01267	0.98733	Hou et al. (2013)
75	10.167	0.01593	0.98407	Hou et al. (2013)
75	13.282	0.01856	0.98144	Hou et al. (2013)
75	16.918	0.01993	0.98007	Hou et al. (2013)
75	6.890	0.01420	0.98580	Bastami et al. (2014)
75	13.790	0.01830	0.98170	Bastami et al. (2014)
80	4.050	0.00800	0.99200	Bamberger et al. (2000)
80	6.060	0.01140	0.98860	Bamberger et al. (2000)

80	7.080	0.01280	0.98720	Bamberger et al. (2000)
80	8.080	0.01400	0.98600	Bamberger et al. (2000)
80	9.090	0.01510	0.98490	Bamberger et al. (2000)
80	10.090	0.01600	0.98400	Bamberger et al. (2000)
80	11.100	0.01720	0.98280	Bamberger et al. (2000)
80	12.100	0.01760	0.98240	Bamberger et al. (2000)
80	13.100	0.01840	0.98160	Bamberger et al. (2000)
80	10.000	0.01724	0.98276	Martin et al. (2009)
80	20.000	0.02170	0.97830	Martin et al. (2009)
80	30.000	0.02327	0.97673	Martin et al. (2009)
100	0.990	0.00175	0.99825	Lucile et al. (2012)
100	2.010	0.00359	0.99641	Lucile et al. (2012)
100	3.050	0.00541	0.99459	Lucile et al. (2012)
100	4.040	0.00697	0.99303	Lucile et al. (2012)
100	5.030	0.00874	0.99126	Lucile et al. (2012)
100	5.030	0.00857	0.99143	Lucile et al. (2012)
100	1.107	0.00169	0.99831	Hou et al. (2013)
100	2.426	0.00390	0.99610	Hou et al. (2013)
100	7.088	0.01085	0.98915	Hou et al. (2013)
100	10.235	0.01365	0.98635	Hou et al. (2013)
100	13.352	0.01626	0.98374	Hou et al. (2013)
100	17.070	0.01872	0.98128	Hou et al. (2013)
100	3.515	0.00697	0.99303	Ahmadi & Chapoy. (2018)
100	7.941	0.01286	0.98714	Ahmadi & Chapoy. (2018)
100	19.340	0.02112	0.97888	Ahmadi & Chapoy. (2018)
100	42.077	0.02578	0.97422	Ahmadi & Chapoy. (2018)
100	8.034	0.01200	0.98800	Mousavi et al. (2024)
102	18.860	0.01920	0.98080	Tong et al. (2013)
102	27.260	0.02190	0.97810	Tong et al. (2013)
102	8.103	0.01220	0.98780	Mousavi et al. (2024)
102	17.586	0.01850	0.98150	Mousavi et al. (2024)
102	40.000	0.02430	0.97570	Mousavi et al. (2024)
120	10.000	0.01335	0.98665	Martin et al. (2009)
120	30.000	0.02458	0.97542	Martin et al. (2009)
120	0.960	0.00141	0.99859	Lucile et al. (2012)
120	2.030	0.00326	0.99674	Lucile et al. (2012)
120	3.050	0.00487	0.99513	Lucile et al. (2012)
120	4.030	0.00617	0.99383	Lucile et al. (2012)
120	4.830	0.00715	0.99285	Lucile et al. (2012)
125	1.158	0.00142	0.99858	Hou et al. (2013)
125	3.176	0.00444	0.99556	Hou et al. (2013)
125	7.321	0.01005	0.98995	Hou et al. (2013)
125	9.984	0.01308	0.98692	Hou et al. (2013)
125	13.456	0.01573	0.98427	Hou et al. (2013)

125	17.435	0.01857	0.98143	Hou et al. (2013)
150	1.428	0.00127	0.99873	Hou et al. (2013)
150	2.987	0.00348	0.99652	Hou et al. (2013)
150	7.197	0.00870	0.99130	Hou et al. (2013)
150	10.014	0.01231	0.98769	Hou et al. (2013)
150	13.418	0.01557	0.98443	Hou et al. (2013)
150	17.355	0.01905	0.98095	Hou et al. (2013)
150	2.631	0.00327	0.99673	Ahmadi & Chapoy. (2018)
150	5.109	0.00665	0.99335	Ahmadi & Chapoy. (2018)
150	9.772	0.01170	0.98830	Ahmadi & Chapoy. (2018)
150	19.549	0.02063	0.97937	Ahmadi & Chapoy. (2018)
150	40.690	0.02839	0.97161	Ahmadi & Chapoy. (2018)
175	1.327	0.00063	0.99937	Hou et al. (2013)
175	7.565	0.00861	0.99139	Hou et al. (2013)
175	3.657	0.00370	0.99630	Hou et al. (2013)
175	10.497	0.01135	0.98865	Hou et al. (2013)
175	13.442	0.01413	0.98587	Hou et al. (2013)
175	17.459	0.01764	0.98236	Hou et al. (2013)

Reference

Ahmadi, P.; Chapoy, A. CO_2 solubility in formation water under sequestration conditions. *Fluid Phase Equilib.* **2018**, *463, 80-90.* https://doi.org/10.1016/j.fluid.2018.02.002.

Anderson, G. K. Solubility of carbon dioxide in water under incipient clathrate formation conditions. *J. Chem.Eng. Data*. **2002**, 47, 219-222. https://doi.org/10.1021/je015518c.

Bamberger, A.; Sieder, G.; Maurer, G. High-pressure (vapor+liquid) equilibrium in binary mixtures of (carbon dioxide+water or acetic acid) at temperatures from 313 to 353 K. *J. Supercrit. Fluids*. **2000**, 17, 97-110. https://doi.org/10.1016/S0896-8446(99)00054-6.

Bando, S.; Takemura, F.; Nishio, M.; Hihara, E.; Akai, M. Solubility of CO_2 in aqueous solutions of NaCl at (30 to 60) C and (10 to 20) MPa. *J. Chem. Eng. Data*. **2003**, 48, 576-579. https://doi.org/10.1021/je0255832.

Bastami, A.; Allahgholi, M.; Pourafshary, P. Experimental and modelling study of the solubility of CO_2 in various $CaCl_2$ solutions at different temperatures and pressures. *Pet. Sci.* **2014**, 11, 569-577. https://doi.org/10.1007/s12182-014-0373-1.

Campos, C. E. P. S.; Villardi, H. G. D. A.; Pessoa, F. L. P.; Uller, A. M. C. Solubility of carbon dioxide in water and hexadecane: Experimental measurement and thermodynamic modeling. *J. Chem. Eng. Data*. **2009**, 54, 2881-2886. https://doi.org/10.1021/je800966f.

Dalmolin, I.; Skovroinski, E.; Biasi, A.; Corazza, M. L.; Dariva, C.; Oliveira, J. V. Solubility of carbon dioxide in binary and ternary mixtures with ethanol and water. *Fluid Phase Equilib*. **2006**, 245, 193-200. https://doi.org/10.1016/j.fluid.2006.04.017.

Dell'era, C.; Uusi-Kyyny, P.; Pokki, J. P.; Pakkanen, M.; Alopaeus, V. Solubility of carbon dioxide in aqueous solutions of diisopropanolamine and methyldiethanolamine. *Fluid Phase Equilib*. **2010**, 293, 101-109. https://doi.org/10.1016/j.fluid.2010.02.035.

Han, J. M.; Shin, H. Y.; Min, B. M.; Han, K. H.; Cho, A. Measurement and correlation of high pressure phase behavior of carbon dioxide+water system. *J. Ind. Eng. Chem*. **2009**, 15, 212-216. https://doi.org/10.1016/j.jiec.2008.09.012.

Hou, S. X.; Maitland, G. C.; Trusler, J. P. M. Measurement and modeling of the phase behavior of the (carbon dioxide+ water) mixture at temperatures from 298.15 K to 448.15 K. *J. Supercrit. Fluids*. **2013**, 73, 87-96. https://doi.org/10.1016/j.supflu.2012.11.011.

Liu, Y.; Hou, M.; Yang, G.; Han, B. Solubility of CO_2 in aqueous solutions of NaCl, KCl, $CaCl_2$ and their mixed salts at different temperatures and pressures. *The Journal of supercritical fluids*, **2011,** 56, 125-129. https://doi.org/10.1016/j.supflu.2010.12.003.

Liu, Y.; Hou, M.; Ning, H.; Yang, D.; Yang, G.; Han, B. Phase equilibria of CO_2+N_2+H_2O and N_2+CO_2+H_2O+NaCl+ KCl+ $CaCl_2$ systems at different temperatures and pressures. *J. Chem. Eng. Data*. **2012**, 57, 1928-1932. https://doi.org/10.1021/je3000958.

Lucile, F.; Cézac, P.; Contamine, F.; Serin, J. P.; Houssin, D.; Arpentinier, P. Solubility of carbon dioxide in water and aqueous solution containing sodium hydroxide at temperatures from (293.15 to 393.15 K) and pressure up to 5 MPa: experimental measurements. *J. Chem. Eng. Data*. **2012** 57, 784-789. https://doi.org/10.1021/je200991x.

Mousavi, R.; Chapoy, A.; Burgass, R. CO_2 solubility in aqueous solution of salts: Experimental study and thermodynamic modelling. *Greenhouse Gas. Sci.Technol.* **2024**, 14, 791-828. https://doi.org/10.1002/ghg.2298.

Muromachi, S.; Shijima, A.; Miyamoto, H.; Ohmura, R. Experimental measurements of carbon dioxide solubility in aqueous tetra-n-butylammonium bromide solutions. *J. Chem. Thermodyn*. **2015**, 85, 94-100. https://doi.org/10.1016/j.jct.2015.01.008.

Ruffine, L.; Trusler, J., P., M. Phase behaviour of mixed-gas hydrate systems containing carbon dioxide. *J. Chem. Thermodyn*. **2010**, 42, 605–611. https://doi.org/10.1016/j.jct.2009.11.019.

Serpa, F. S.; Vidal, R. S.; Filho, J. H. B. A.; Nascimento, J. F. D.; Ciambelli, J. R. P.; Figueiredo, C. M. S.; Salazar-Banda, G. R.; Santos, A. F.; Fortuny, M.; Franceschi, E.; Dariva, C. Solubility of carbon dioxide in ethane-1, 2-diol–water mixtures. *J. Chem. Eng. Data*. **2013**, 58, 3464-3469. https://doi.org/10.1021/je400736w.

Servio, P.; Englezos, P. Effect of temperature and pressure on the solubility of carbon dioxide in water in the presence of gas hydrate. *Fluid Phase Equilib*. **2001**, 190, 127-134. https://doi.org/10.1016/S0378-3812(01)00598-2.

Tang, Y.; Bian, X.; Du, Z.; Wang, C. Measurement and prediction model of carbon dioxide solubility in aqueous solutions containing bicarbonate anion. *Fluid Phase Equilib*. **2015**, 386, 56-64. https://doi.org/10.1016/j.fluid.2014.11.025.

Tong, D.; Trusler, J. P. M.; Vega-Maza, D. Solubility of CO_2 in aqueous solutions of $CaCl_2$ or $MgCl_2$ and in a synthetic;c formation brine at temperatures up to 423 K and pressures up to 40 MPa. *J. Chem. Eng. Data*. **2013**, 58, 2116-2124. https://doi.org/10.1021/je400396s.

Valtz, A.; Chapoy, A.; Coquelet, C.; Paricaud, P.; Richon, D. Vapour–liquid equilibria in the carbon dioxide–water system, measurement and modelling from 278.2 to 318.2 K. *Fluid Phase Equilib*. **2004**, 226, 333-344. https://doi.org/10.1016/j.fluid.2004.10.013.

Zhang, G.; Wu, Y.; Ma, P.; Wu, G.; Li, D. Measurement and correlation of solubility of carbon monoxide and other gases solubility in phenol. *J. Chem. Ind. Eng. (China)* **2005**, 56, 2039.
